# Distance learning and telemedicine in the area of Otorhinolaryngology: lessons in times of pandemic^☆^

**DOI:** 10.1016/j.bjorl.2020.03.003

**Published:** 2020-04-06

**Authors:** Almiro José Machado Júnior, Henrique Furlan Pauna

**Affiliations:** Universidade Estadual de Campinas (Unicamp), Disciplina de Otorrinolaringologia, Cirurgia de Cabeça e Pescoço, Campinas, SP, Brazil

As COVID-19 is identified worldwide, governments have imposed quarantines and travel restrictions on an unprecedented scale. Countries such as China, Italy, Spain and the United States have imposed restrictions on their citizens or immigrants. Still, the number of cases and deaths continues to increase.^1^ Quarantines and travel bans are often the first response to new infectious diseases. However, these old tools are usually of limited use for highly communicable diseases.^1^

Currently, the discussion about the COVID-19 pandemic imposes restrictions on the mobilization of students (including in the health area), with classes being canceled and the teaching hospitals operating with “on duty” shifts only.^2^ Flattening the curve of the number of new cases–slowing the spread of COVID-19 through space and time–is crucial. The health system cannot sustain a massive influx of infectious cases into emergency units and hospitals. Patients with mild symptoms should stay home whenever possible.

Recognizing that many patients need guidance or recommendations, the use of virtual guidance services that have already been implemented or are under development can be an alternative to face-to-face visits to hospitals or medical offices.^3^ A strategy that is starting to be implemented is telemedicine to the consumer, an approach that helps with efficient direct screening, helping to maintain the quarantine and protecting doctors and patients. Telemedicine allows doctors and patients to communicate using smartphones or webcam-enabled computers. Respiratory symptoms–admittedly COVID-19–are among the conditions most commonly assessed through this approach. Many medical decisions are cognitive ones, and telemedicine can provide quick access to other medical specialties that are not immediately available in person. Moreover, healthcare providers can easily obtain detailed travel and exposure histories and use algorithms to standardize guidance and information.

Similarly, distance learning (DL) is a method that has been widely used as a tool in helping to disseminate culture and knowledge. Many countries have benefited from this tool, and in Brazil, a country of continental dimensions, this modality has been shown to be an effective method of spreading knowledge, considering the DL figures throughout the country.^4^ A recurring discussion in the mass and specialized media is the use of distance learning in the health area. Professional class entities in the health area have questioned and even opposed the use of DL as a training method in undergraduate courses carried out entirely at distance, although other areas are benefiting from this tool.

There is a wide range of tools used in DL among the researched articles: they range from online tools, use of tablets and applications for smartphones, to 3D-anatomical pieces. However, a condition is recurrent in these studies: these tools are used as a complement in the teaching-learning process and do not take away the teacher's role in this relationship.^5^

The successful use of DL in medical education requires it to meet the needs of students and it must be aligned with the program and the contexts in which it is used. Researchers have noted that individual differences can also play an important role in the effectiveness of these tools. Students preferred e-learning technologies and perform better with digital media systems, because they use active approaches and make better transfer of concepts in new situations.^4^

Although several studies point to new paths regarding the use of virtual tools for patient guidance or teaching-learning, the limits to which the medical content should be made available are not known. Currently, in Brazil, there is a great discussion about the non-use of DL in undergraduate courses in the health area; however, among the reviewed articles, more than half show successful DL experiences in undergraduate medical courses (and also in the area of otorhinolaryngology), not only in theoretical disciplines, but also in practical and surgical ones. These studies use DL as a complementary tool in the teaching-learning process. However, how many hours and how much content should be transmitted in this modality? Should we rethink the teaching-learning dynamic? Will the teacher's role as a transmitter of knowledge be limited to the advent of distance learning? New studies must be carried out to evaluate these variables.

Disasters and pandemics represent unique challenges to education and health care services. Although telemedicine does not solve all of them, it is adequate for scenarios where the infrastructure remains intact and doctors are available to assist patients. Payment and regulatory structures, state licensing, hospital accreditation and program implementation take time to be completed, but health systems that have already invested in telemedicine are well established to ensure that patients with COVID-19 receive the care they need.

## Conflicts of interest

The authors declare no conflicts of interest.
